# 5-Methyl-*N*′-[(3*Z*)-2-oxo-2,3-dihydro-1*H*-indol-3-yl­idene]-1-phenyl-1*H*-1,2,3-triazole-4-carbohydrazide

**DOI:** 10.1107/S1600536813007502

**Published:** 2013-03-23

**Authors:** Hanan A. Mohamed, Bakr F. Abdel-Wahab, Seik Weng Ng, Edward R. T. Tiekink

**Affiliations:** a Applied Organic Chemistry Department, National Research Centre, Dokki, 12622 Giza, Egypt; b Department of Chemistry, University of Malaya, 50603 Kuala Lumpur, Malaysia; c Chemistry Department, Faculty of Science, King Abdulaziz University, PO Box 80203 Jeddah, Saudi Arabia

## Abstract

In the title compound, C_18_H_14_N_6_O_2_, the benzene ring is slightly twisted out of the plane of the 1,2,3-triazole ring (r.m.s. deviation = 0.010 Å), forming a dihedral angle of 6.20 (13)°. The nine non-H ring atoms of the fused five- and six-membered ring system are almost coplanar (r.m.s. deviation = 0.032 Å). The near coplanarity in the central residue is consolidated by an intra­molecular bifurcated N—H⋯(O,N) hydrogen bond. The conformation about the N=C bond is *Z*. In the crystal, supra­molecular chains along [101] are sustained by N—H⋯O hydrogen bonds and C—H⋯O inter­actions. These are consolidated into a three-dimensional architecture by C—H⋯π and π–π inter­actions; the latter occur between centrosymmetrically related 1,2,3-triazole rings [centroid–centroid distance = 3.6056 (14) Å].

## Related literature

For the biological activity of 1,2,3-triazoles, see: Abdel-Wahab *et al.* (2012 ▶); Jordão *et al.* (2011 ▶).
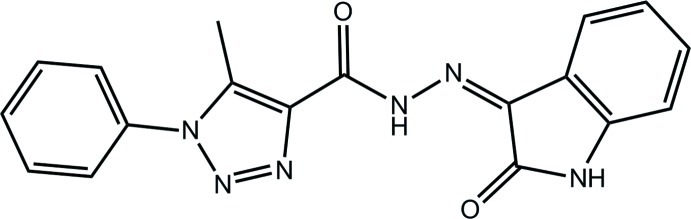


## Experimental

### 

#### Crystal data

C_18_H_14_N_6_O_2_
*M* *_r_* = 346.35
Monoclinic, 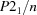

*a* = 7.1835 (8) Å
*b* = 18.620 (2) Å
*c* = 12.2949 (11) Å
β = 101.676 (11)°
*V* = 1610.5 (3) Å^3^
*Z* = 4
Mo *K*α radiation
μ = 0.10 mm^−1^
*T* = 295 K
0.25 × 0.05 × 0.05 mm

#### Data collection

Agilent SuperNova Dual diffractometer with an Atlas detector
Absorption correction: multi-scan (*CrysAlis PRO*; Agilent, 2011 ▶) *T*_min_ = 0.971, *T*_max_ = 1.000
8876 measured reflections
3715 independent reflections
2131 reflections with *I* > 2σ(*I*)
*R*_int_ = 0.039

#### Refinement

*R*[*F* ^2^ > 2σ(*F* ^2^)] = 0.058
*wR*(*F* ^2^) = 0.147
*S* = 1.03
3715 reflections
244 parameters
2 restraints
H atoms treated by a mixture of independent and constrained refinement
Δρ_max_ = 0.16 e Å^−3^
Δρ_min_ = −0.20 e Å^−3^

### 

Data collection: *CrysAlis PRO* (Agilent, 2011 ▶); cell refinement: *CrysAlis PRO*; data reduction: *CrysAlis PRO*; program(s) used to solve structure: *SHELXS97* (Sheldrick, 2008 ▶); program(s) used to refine structure: *SHELXL97* (Sheldrick, 2008 ▶); molecular graphics: *ORTEP-3 for Windows* (Farrugia, 2012 ▶) and *DIAMOND* (Brandenburg, 2006 ▶); software used to prepare material for publication: *publCIF* (Westrip, 2010 ▶).

## Supplementary Material

Crystal structure: contains datablock(s) global, I. DOI: 10.1107/S1600536813007502/hb7059sup1.cif

Structure factors: contains datablock(s) I. DOI: 10.1107/S1600536813007502/hb7059Isup2.hkl

Supplementary material file. DOI: 10.1107/S1600536813007502/hb7059Isup3.cml

Additional supplementary materials:  crystallographic information; 3D view; checkCIF report

## Figures and Tables

**Table 1:** Hydrogen-bond geometry (Å, °) *Cg*1 is the centroid of the C13–C18 ring.

*D*—H⋯*A*	*D*—H	H⋯*A*	*D*⋯*A*	*D*—H⋯*A*
N4—H4⋯O2	0.89 (1)	1.95 (2)	2.685 (2)	138 (2)
N4—H4⋯N3	0.89 (1)	2.34 (2)	2.715 (3)	105 (2)
N6—H6⋯O1^i^	0.88 (1)	1.95 (2)	2.783 (2)	157 (3)
C14—H14⋯O2^ii^	0.93	2.33	3.244 (3)	168
C9—H9*C*⋯*Cg*1^iii^	0.96	2.94	3.822 (2)	154

Symmetry codes: (i) 
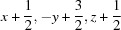
; (ii) 
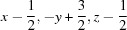
; (iii) 
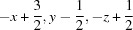
.

**Fig. 1.**
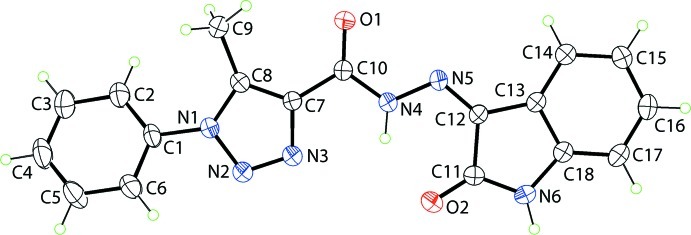
The molecular structure of (I) showing displacement ellipsoids at the 35% probability level.

**Fig. 2.**
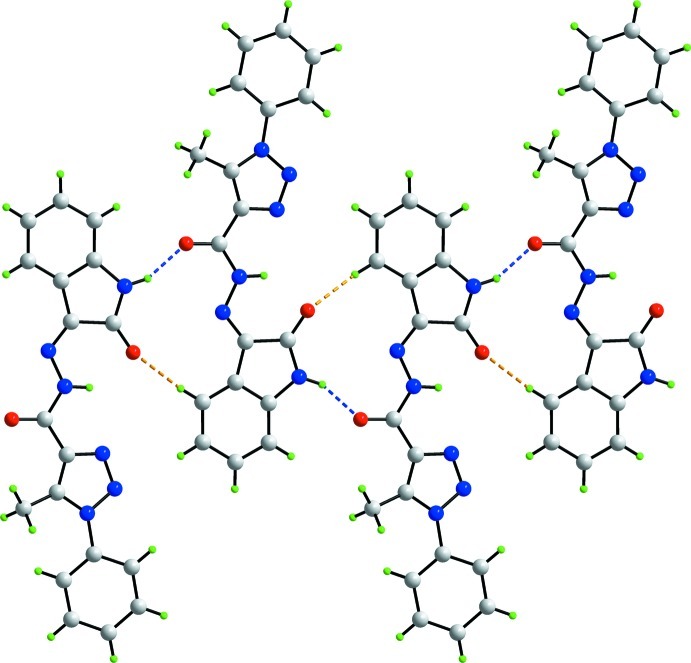
A view of the supramolecular chain along [1 0 1] sustained by N—H···O and C—H···O interactions, shown as blue and orange dashed lines, respectively.

**Fig. 3.**
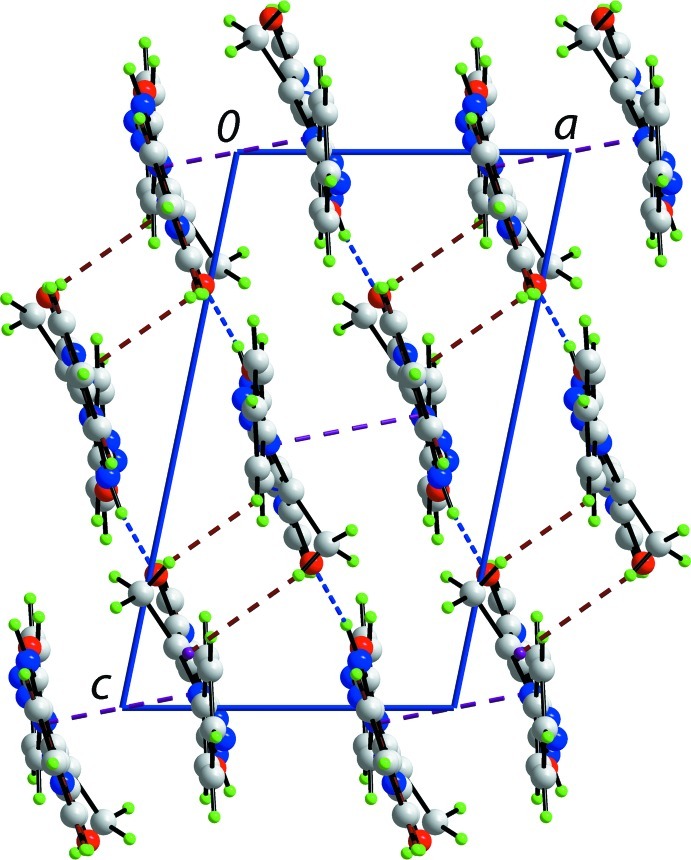
A view of the crystal packing in projection down the b axis. The N—H···O, C—H···π and π—π interactions are shown as blue, brown and purple dashed lines, respectively.

## supplementary crystallographic information

### Comment

The title compound (I) was investigated in relation to the established biological activities exhibited by 1,2,3-triazoles (Abdel-Wahab *et al.*, 2012; Jordão *et al.*, 2011).

With respect to the 1,2,3-triazole ring (r.m.s. deviation = 0.010 Å) in (I), Fig. 1, both the benzene [dihedral angle = 26.20 (13)°] and to a lesser extent the amide residues are twisted [C10—C7—C8—C9 torsion angle = -8.8 (4)°]. The nine non-hydrogen ring atoms of the 3-imino-1*H*-indol-2-one residue are co-planar, having a r.m.s. deviation of 0.032 Å. The formation of intramolecular N4—H···O2 and N4—H···N3 hydrogen bonds, Table 1, confers stability to the co-planar arrangement in the central region of the molecule, *i.e*. the N4—N5—C12—C11 and N3—C7—C10—N4 torsion angles are -0.9 (3) and -6.3 (3)°, respectively. Finally, the conformation about the N5═C12 bond is *Z*.

Supramolecular chains propagating along [1 0 1] feature in the crystal packing. These are sustained by N6—H···O1 hydrogen bonds and supported by C14—H···O2 interactions, Table 1, and lead to 12-membered {···OCN_2_C_3_H···OCNH} synthons, Fig. 2. The chains assemble in layers parallel to (1 0 1) and are connected into the three-dimensional architecture by C—H···π interactions between a methyl-H and the C13–C18 benzene ring, Table 1, and π—π interactions between centrosymmetrically related 1,2,3-triazole rings [inter-centroid distance = 3.6056 (14) Å for symmetry operation 1 - *x*, 1 - *y*, 1 - *z*], Fig. 3.

### Experimental

A mixture of 5-methyl-1-phenyl-1*H*-1,2,3-triazole-4-carbohydrazide (0.22 g, 0.001 *M*) and indoline-2,3-dione (0.15 g, 0.001 *M*) in anhydrous ethanol (30 ml) in the presence of a catalytic amount of glacial acetic acid was heated under reflux for about 4 h. The resultant solid was filtered and dried. Re-crystallization was by slow evaporation of its DMF solution which yielded yellow prisms in 73% yield; *M*.pt: 562–564 K.

### Refinement

Carbon-bound H-atoms were placed in calculated positions (C—H = 0.93 to 0.96 Å) and were included in the refinement in the riding model approximation, with *U*_iso_(H) = 1.2–1.5*U*_equiv_(C). Nitrogen-bond H-atoms were refined with the distance restraint N—H = 0.88±0.01 Å, and with unrestricted *U*_iso_.

### Crystal data

**Table:**

C_18_H_14_N_6_O_2_	*F*(000) = 720
*M**_r_* = 346.35	*D*_x_ = 1.428 Mg m^−^^3^
Monoclinic, *P*2_1_/*n*	Mo *K*α radiation, λ = 0.71073 Å
Hall symbol: -P 2yn	Cell parameters from 1697 reflections
*a* = 7.1835 (8) Å	θ = 2.9–27.5°
*b* = 18.620 (2) Å	µ = 0.10 mm^−^^1^
*c* = 12.2949 (11) Å	*T* = 295 K
β = 101.676 (11)°	Prism, yellow
*V* = 1610.5 (3) Å^3^	0.25 × 0.05 × 0.05 mm
*Z* = 4	

### Data collection

**Table:**

Agilent SuperNova Dual diffractometer with an Atlas detector	3715 independent reflections
Radiation source: SuperNova (Mo) X-ray Source	2131 reflections with *I* > 2σ(*I*)
Mirror monochromator	*R*_int_ = 0.039
Detector resolution: 10.4041 pixels mm^-1^	θ_max_ = 27.6°, θ_min_ = 3.0°
ω scan	*h* = −9→9
Absorption correction: multi-scan (*CrysAlis PRO*; Agilent, 2011)	*k* = −21→24
*T*_min_ = 0.971, *T*_max_ = 1.000	*l* = −15→16
8876 measured reflections	

### Refinement

**Table:**

Refinement on *F*^2^	Primary atom site location: structure-invariant direct methods
Least-squares matrix: full	Secondary atom site location: difference Fourier map
*R*[*F*^2^ > 2σ(*F*^2^)] = 0.058	Hydrogen site location: inferred from neighbouring sites
*wR*(*F*^2^) = 0.147	H atoms treated by a mixture of independent and constrained refinement
*S* = 1.03	*w* = 1/[σ^2^(*F*_o_^2^) + (0.0482*P*)^2^ + 0.2228*P*] where *P* = (*F*_o_^2^ + 2*F*_c_^2^)/3
3715 reflections	(Δ/σ)_max_ < 0.001
244 parameters	Δρ_max_ = 0.16 e Å^−^^3^
2 restraints	Δρ_min_ = −0.20 e Å^−^^3^

### Special details

**Table:**

Geometry. All e.s.d.'s (except the e.s.d. in the dihedral angle between two l.s. planes) are estimated using the full covariance matrix. The cell e.s.d.'s are taken into account individually in the estimation of e.s.d.'s in distances, angles and torsion angles; correlations between e.s.d.'s in cell parameters are only used when they are defined by crystal symmetry. An approximate (isotropic) treatment of cell e.s.d.'s is used for estimating e.s.d.'s involving l.s. planes.
Refinement. Refinement of *F*^2^ against ALL reflections. The weighted *R*-factor *wR* and goodness of fit *S* are based on *F*^2^, conventional *R*-factors *R* are based on *F*, with *F* set to zero for negative *F*^2^. The threshold expression of *F*^2^ > σ(*F*^2^) is used only for calculating *R*-factors(gt) *etc*. and is not relevant to the choice of reflections for refinement. *R*-factors based on *F*^2^ are statistically about twice as large as those based on *F*, and *R*- factors based on ALL data will be even larger.

### Fractional atomic coordinates and isotropic or equivalent isotropic displacement parameters (Å^2^)

**Table:**

	*x*	*y*	*z*	*U*_iso_*/*U*_eq_	
O1	0.5195 (3)	0.58729 (9)	0.26022 (12)	0.0597 (5)	
O2	0.8162 (3)	0.71122 (10)	0.60828 (12)	0.0613 (5)	
N1	0.7254 (2)	0.41347 (10)	0.47664 (13)	0.0418 (5)	
N2	0.8247 (3)	0.45889 (11)	0.55553 (14)	0.0485 (5)	
N3	0.7912 (3)	0.52406 (11)	0.52030 (14)	0.0461 (5)	
N4	0.6750 (3)	0.64843 (10)	0.41239 (14)	0.0446 (5)	
N5	0.6338 (3)	0.71428 (10)	0.36535 (14)	0.0429 (5)	
N6	0.8130 (3)	0.83398 (12)	0.58399 (15)	0.0517 (5)	
C1	0.7454 (3)	0.33768 (13)	0.49580 (18)	0.0430 (6)	
C2	0.7219 (4)	0.28982 (14)	0.4094 (2)	0.0553 (7)	
H2	0.6931	0.3062	0.3365	0.066*	
C3	0.7413 (4)	0.21701 (15)	0.4314 (2)	0.0655 (8)	
H3	0.7229	0.1844	0.3729	0.079*	
C4	0.7873 (4)	0.19263 (16)	0.5383 (3)	0.0670 (8)	
H4A	0.8008	0.1437	0.5527	0.080*	
C5	0.8131 (4)	0.24064 (16)	0.6236 (2)	0.0669 (8)	
H5	0.8457	0.2240	0.6962	0.080*	
C6	0.7917 (4)	0.31390 (15)	0.60440 (19)	0.0558 (7)	
H6A	0.8081	0.3462	0.6632	0.067*	
C7	0.6714 (3)	0.52181 (12)	0.41830 (16)	0.0390 (5)	
C8	0.6253 (3)	0.45209 (13)	0.39001 (16)	0.0397 (5)	
C9	0.4854 (3)	0.42228 (13)	0.29457 (18)	0.0509 (6)	
H9A	0.3960	0.4590	0.2642	0.076*	
H9B	0.4190	0.3828	0.3195	0.076*	
H9C	0.5509	0.4059	0.2385	0.076*	
C10	0.6140 (3)	0.58757 (12)	0.35507 (17)	0.0416 (6)	
C11	0.7793 (3)	0.76558 (13)	0.55151 (18)	0.0466 (6)	
C12	0.6838 (3)	0.76850 (12)	0.43033 (16)	0.0395 (5)	
C13	0.6616 (3)	0.84409 (12)	0.40176 (16)	0.0392 (5)	
C14	0.5752 (3)	0.88056 (13)	0.30717 (17)	0.0464 (6)	
H14	0.5144	0.8557	0.2443	0.056*	
C15	0.5807 (3)	0.95496 (14)	0.30778 (19)	0.0531 (7)	
H15	0.5234	0.9804	0.2447	0.064*	
C16	0.6707 (4)	0.99165 (14)	0.4013 (2)	0.0595 (7)	
H16	0.6748	1.0416	0.3997	0.071*	
C17	0.7551 (4)	0.95587 (14)	0.49753 (19)	0.0565 (7)	
H17	0.8150	0.9809	0.5604	0.068*	
C18	0.7469 (3)	0.88237 (14)	0.49662 (17)	0.0438 (6)	
H4	0.739 (3)	0.6463 (14)	0.4827 (10)	0.063 (8)*	
H6	0.876 (4)	0.8480 (15)	0.6497 (13)	0.087 (10)*	

### Atomic displacement parameters (Å^2^)

**Table:**

	*U*^11^	*U*^22^	*U*^33^	*U*^12^	*U*^13^	*U*^23^
O1	0.0920 (12)	0.0383 (11)	0.0371 (8)	0.0049 (9)	−0.0148 (8)	−0.0004 (7)
O2	0.0829 (12)	0.0428 (11)	0.0470 (9)	−0.0027 (10)	−0.0136 (8)	0.0080 (8)
N1	0.0514 (11)	0.0315 (11)	0.0394 (9)	0.0026 (9)	0.0015 (8)	0.0026 (8)
N2	0.0584 (12)	0.0370 (12)	0.0436 (10)	0.0009 (10)	−0.0049 (9)	0.0016 (9)
N3	0.0559 (11)	0.0359 (12)	0.0417 (10)	0.0019 (10)	−0.0020 (9)	0.0023 (8)
N4	0.0587 (11)	0.0312 (12)	0.0382 (10)	0.0008 (10)	−0.0041 (9)	0.0025 (8)
N5	0.0538 (11)	0.0290 (11)	0.0416 (10)	0.0017 (9)	−0.0004 (8)	0.0019 (8)
N6	0.0685 (13)	0.0405 (13)	0.0380 (10)	−0.0032 (11)	−0.0087 (9)	−0.0057 (9)
C1	0.0489 (12)	0.0282 (13)	0.0512 (13)	0.0054 (11)	0.0087 (10)	0.0081 (10)
C2	0.0716 (16)	0.0370 (16)	0.0564 (14)	0.0058 (14)	0.0105 (12)	0.0031 (12)
C3	0.0800 (19)	0.0370 (16)	0.0777 (18)	0.0060 (15)	0.0119 (15)	−0.0003 (14)
C4	0.0746 (18)	0.0349 (16)	0.090 (2)	0.0088 (14)	0.0119 (16)	0.0152 (15)
C5	0.0797 (19)	0.0528 (19)	0.0660 (16)	0.0160 (16)	0.0095 (14)	0.0251 (15)
C6	0.0688 (16)	0.0438 (16)	0.0517 (14)	0.0122 (14)	0.0051 (12)	0.0096 (12)
C7	0.0468 (12)	0.0318 (13)	0.0350 (10)	0.0039 (10)	0.0005 (9)	−0.0010 (9)
C8	0.0458 (12)	0.0350 (14)	0.0364 (11)	0.0028 (11)	0.0041 (9)	0.0036 (10)
C9	0.0614 (14)	0.0355 (14)	0.0495 (13)	−0.0020 (12)	−0.0038 (11)	−0.0025 (11)
C10	0.0505 (12)	0.0317 (13)	0.0395 (11)	0.0029 (11)	0.0018 (10)	−0.0014 (10)
C11	0.0546 (13)	0.0393 (15)	0.0399 (12)	−0.0006 (12)	−0.0047 (10)	0.0005 (11)
C12	0.0438 (12)	0.0315 (13)	0.0387 (11)	−0.0017 (10)	−0.0021 (9)	−0.0024 (10)
C13	0.0440 (11)	0.0333 (13)	0.0379 (11)	−0.0030 (11)	0.0026 (9)	−0.0032 (9)
C14	0.0548 (13)	0.0380 (14)	0.0422 (12)	−0.0026 (12)	0.0003 (10)	−0.0018 (11)
C15	0.0683 (16)	0.0372 (15)	0.0491 (13)	0.0008 (13)	0.0003 (12)	0.0063 (11)
C16	0.0813 (18)	0.0311 (14)	0.0621 (16)	−0.0035 (14)	0.0051 (14)	−0.0005 (12)
C17	0.0749 (17)	0.0382 (15)	0.0510 (14)	−0.0083 (14)	−0.0004 (12)	−0.0101 (12)
C18	0.0532 (13)	0.0348 (14)	0.0402 (11)	−0.0026 (11)	0.0022 (10)	−0.0019 (10)

### Geometric parameters (Å, º)

**Table:**

O1—C10	1.225 (2)	C5—C6	1.388 (4)
O2—C11	1.228 (3)	C5—H5	0.9300
N1—C8	1.363 (2)	C6—H6A	0.9300
N1—N2	1.373 (2)	C7—C8	1.367 (3)
N1—C1	1.433 (3)	C7—C10	1.464 (3)
N2—N3	1.294 (3)	C8—C9	1.490 (3)
N3—C7	1.370 (2)	C9—H9A	0.9600
N4—C10	1.359 (3)	C9—H9B	0.9600
N4—N5	1.363 (2)	C9—H9C	0.9600
N4—H4	0.893 (10)	C11—C12	1.510 (3)
N5—C12	1.292 (3)	C12—C13	1.452 (3)
N6—C11	1.342 (3)	C13—C14	1.381 (3)
N6—C18	1.409 (3)	C13—C18	1.398 (3)
N6—H6	0.881 (10)	C14—C15	1.386 (3)
C1—C2	1.370 (3)	C14—H14	0.9300
C1—C6	1.382 (3)	C15—C16	1.381 (3)
C2—C3	1.384 (3)	C15—H15	0.9300
C2—H2	0.9300	C16—C17	1.386 (3)
C3—C4	1.366 (4)	C16—H16	0.9300
C3—H3	0.9300	C17—C18	1.370 (4)
C4—C5	1.362 (4)	C17—H17	0.9300
C4—H4A	0.9300		
			
C8—N1—N2	110.09 (18)	C7—C8—C9	130.12 (19)
C8—N1—C1	131.86 (18)	C8—C9—H9A	109.5
N2—N1—C1	118.04 (16)	C8—C9—H9B	109.5
N3—N2—N1	107.84 (15)	H9A—C9—H9B	109.5
N2—N3—C7	108.50 (18)	C8—C9—H9C	109.5
C10—N4—N5	120.71 (17)	H9A—C9—H9C	109.5
C10—N4—H4	120.9 (17)	H9B—C9—H9C	109.5
N5—N4—H4	118.4 (17)	O1—C10—N4	123.7 (2)
C12—N5—N4	115.54 (17)	O1—C10—C7	123.0 (2)
C11—N6—C18	111.56 (17)	N4—C10—C7	113.29 (17)
C11—N6—H6	126 (2)	O2—C11—N6	127.5 (2)
C18—N6—H6	123 (2)	O2—C11—C12	126.4 (2)
C2—C1—C6	120.5 (2)	N6—C11—C12	106.15 (19)
C2—C1—N1	121.36 (19)	N5—C12—C13	127.19 (18)
C6—C1—N1	118.1 (2)	N5—C12—C11	126.6 (2)
C1—C2—C3	119.6 (2)	C13—C12—C11	106.22 (18)
C1—C2—H2	120.2	C14—C13—C18	119.9 (2)
C3—C2—H2	120.2	C14—C13—C12	133.57 (19)
C4—C3—C2	120.6 (3)	C18—C13—C12	106.52 (17)
C4—C3—H3	119.7	C13—C14—C15	118.6 (2)
C2—C3—H3	119.7	C13—C14—H14	120.7
C5—C4—C3	119.4 (3)	C15—C14—H14	120.7
C5—C4—H4A	120.3	C16—C15—C14	120.5 (2)
C3—C4—H4A	120.3	C16—C15—H15	119.8
C4—C5—C6	121.4 (2)	C14—C15—H15	119.8
C4—C5—H5	119.3	C15—C16—C17	121.6 (2)
C6—C5—H5	119.3	C15—C16—H16	119.2
C1—C6—C5	118.4 (3)	C17—C16—H16	119.2
C1—C6—H6A	120.8	C18—C17—C16	117.6 (2)
C5—C6—H6A	120.8	C18—C17—H17	121.2
C8—C7—N3	109.67 (19)	C16—C17—H17	121.2
C8—C7—C10	129.15 (18)	C17—C18—C13	121.8 (2)
N3—C7—C10	121.2 (2)	C17—C18—N6	128.7 (2)
N1—C8—C7	103.88 (17)	C13—C18—N6	109.5 (2)
N1—C8—C9	125.7 (2)		
			
C8—N1—N2—N3	−0.7 (3)	N3—C7—C10—O1	174.1 (2)
C1—N1—N2—N3	178.00 (19)	C8—C7—C10—N4	175.3 (2)
N1—N2—N3—C7	−0.4 (2)	N3—C7—C10—N4	−6.3 (3)
C10—N4—N5—C12	174.1 (2)	C18—N6—C11—O2	−178.5 (2)
C8—N1—C1—C2	25.8 (4)	C18—N6—C11—C12	0.8 (3)
N2—N1—C1—C2	−152.5 (2)	N4—N5—C12—C13	178.4 (2)
C8—N1—C1—C6	−155.0 (2)	N4—N5—C12—C11	−0.9 (3)
N2—N1—C1—C6	26.7 (3)	O2—C11—C12—N5	−3.6 (4)
C6—C1—C2—C3	1.2 (4)	N6—C11—C12—N5	177.0 (2)
N1—C1—C2—C3	−179.6 (2)	O2—C11—C12—C13	176.9 (2)
C1—C2—C3—C4	−1.3 (4)	N6—C11—C12—C13	−2.4 (3)
C2—C3—C4—C5	0.3 (4)	N5—C12—C13—C14	5.4 (4)
C3—C4—C5—C6	0.7 (4)	C11—C12—C13—C14	−175.2 (2)
C2—C1—C6—C5	−0.2 (4)	N5—C12—C13—C18	−176.4 (2)
N1—C1—C6—C5	−179.4 (2)	C11—C12—C13—C18	3.1 (2)
C4—C5—C6—C1	−0.8 (4)	C18—C13—C14—C15	2.1 (3)
N2—N3—C7—C8	1.3 (3)	C12—C13—C14—C15	−179.9 (2)
N2—N3—C7—C10	−177.4 (2)	C13—C14—C15—C16	−0.1 (4)
N2—N1—C8—C7	1.4 (2)	C14—C15—C16—C17	−1.1 (4)
C1—N1—C8—C7	−177.0 (2)	C15—C16—C17—C18	0.3 (4)
N2—N1—C8—C9	−173.2 (2)	C16—C17—C18—C13	1.7 (4)
C1—N1—C8—C9	8.4 (4)	C16—C17—C18—N6	−176.8 (2)
N3—C7—C8—N1	−1.6 (2)	C14—C13—C18—C17	−2.9 (4)
C10—C7—C8—N1	176.9 (2)	C12—C13—C18—C17	178.5 (2)
N3—C7—C8—C9	172.6 (2)	C14—C13—C18—N6	175.9 (2)
C10—C7—C8—C9	−8.8 (4)	C12—C13—C18—N6	−2.7 (3)
N5—N4—C10—O1	−0.5 (4)	C11—N6—C18—C17	179.9 (3)
N5—N4—C10—C7	179.92 (19)	C11—N6—C18—C13	1.2 (3)
C8—C7—C10—O1	−4.3 (4)		

### Hydrogen-bond geometry (Å, º)

Cg1 is the centroid of the C13–C18 ring.

**Table:**

*D*—H···*A*	*D*—H	H···*A*	*D*···*A*	*D*—H···*A*
N4—H4···O2	0.89 (1)	1.95 (2)	2.685 (2)	138 (2)
N4—H4···N3	0.89 (1)	2.34 (2)	2.715 (3)	105 (2)
N6—H6···O1^i^	0.88 (1)	1.95 (2)	2.783 (2)	157 (3)
C14—H14···O2^ii^	0.93	2.33	3.244 (3)	168
C9—H9*C*···*Cg*1^iii^	0.96	2.94	3.822 (2)	154

Symmetry codes: (i) *x*+1/2, −*y*+3/2, *z*+1/2; (ii) *x*−1/2, −*y*+3/2, *z*−1/2; (iii) −*x*+3/2, *y*−1/2, −*z*+1/2.
